# ADAM and ADAMTS Family Proteins and Snake Venom Metalloproteinases: A Structural Overview

**DOI:** 10.3390/toxins8050155

**Published:** 2016-05-17

**Authors:** Soichi Takeda

**Affiliations:** Department of Cardiac Physiology, National Cerebral and Cardiovascular Center Research Institute, 5-7-1, Fujishirodai, Suita, Osaka 565-8565, Japan; stakeda@ri.ncvc.go.jp; Tel.: +81-6-6833-5012

**Keywords:** snake venom, metalloproteinase, disintegrin, ADAM, ADAMTS, MDC, reprolysin, adamalysin, shedding, crystal structure

## Abstract

A disintegrin and metalloproteinase (ADAM) family proteins constitute a major class of membrane-anchored multidomain proteinases that are responsible for the shedding of cell-surface protein ectodomains, including the latent forms of growth factors, cytokines, receptors and other molecules. Snake venom metalloproteinases (SVMPs) are major components in most viper venoms. SVMPs are primarily responsible for hemorrhagic activity and may also interfere with the hemostatic system in envenomed animals. SVMPs are phylogenetically most closely related to ADAMs and, together with ADAMs and related ADAM with thrombospondin motifs (ADAMTS) family proteinases, constitute adamalysins/reprolysins or the M12B clan (MEROPS database) of metalloproteinases. Although the catalytic domain structure is topologically similar to that of other metalloproteinases such as matrix metalloproteinases, the M12B proteinases have a modular structure with multiple non-catalytic ancillary domains that are not found in other proteinases. Notably, crystallographic studies revealed that, in addition to the conserved metalloproteinase domain, M12B members share a hallmark cysteine-rich domain designated as the “ADAM_CR” domain. Despite their name, ADAMTSs lack disintegrin-like structures and instead comprise two ADAM_CR domains. This review highlights the current state of our knowledge on the three-dimensional structures of M12B proteinases, focusing on their unique domains that may collaboratively participate in directing these proteinases to specific substrates.

## 1. Introduction

A disintegrin and metalloproteinase (ADAM) family proteins, also known as metalloproteinase-disintegrins or metalloproteinase/disintegrin-like/cysteine-rich (MDC) proteins, are type-I transmembrane and soluble glycoproteins that have diverse functions in cell adhesion, migration, proteolysis and signaling [1,2,3]. The best-characterized function of the membrane-anchored ADAMs is their involvement in ectodomain shedding of various cell-surface proteins, including the latent forms of growth factors, cytokines and their receptors and cell-adhesion molecules. For example, ADAM17 (TACE, TNF-α converting enzyme) is a sheddase involved in the processing of tumor necrosis factor-α [4,5] and a broad range of other cell-surface molecules [1]. Identification of a patient lacking ADAM17 revealed that ADAM17 is involved in the protection of the skin and intestinal barrier [6]. Another major family member, ADAM10, is a principal player in signaling via the Notch and Eph/ephrin pathways [7]. ADAMs play key roles in normal development and morphogenesis. Dysregulation of shedding activity is a crucial factor in a number of pathologies, such as inflammation, neurodegenerative disease, cardiovascular disease, asthma, cancer and others [1,3,8,9,10,11]. So far, 40 family members have been identified in the mammalian genome, of which 37 are expressed in mice (most of them in a testis-specific manner) and 20, excluding presumed pseudogenes, are expressed in humans [3]. However, only 12 of the human ADAM members (ADAM8, 9, 10, 12, 15, 17, 19, 20, 21, 28, 30 and 33) contain a functional catalytic consensus sequence (HEXGEHXXGXXH, see below). The physiological function of the proteinase-inactive ADAMs (ADAM2, 7, 11, 18, 22, 23, 29 and 32) remains largely unknown, although some members of this group play important roles in development and function as adhesion molecules rather than proteinases [12,13]. ADAMs are widely expressed in mammalian tissues, and the observed phenotypes of ADAM knockout mice are subsequently diverse, although only ADAM10, 17 and 19 are essential for mouse development [3]. An increasing number of transmembrane proteins have been identified as the targets of ADAM-mediated proteolysis [1]. Some of these substrates can be cleaved by different ADAMs while others appear to be specific to an individual ADAM. In addition, no clear consensus sequences have so far been identified around the scissile bonds of the ADAM substrates. These observations highlight the need for a better understanding of how the substrate specificity and proteolytic activity of ADAMs are determined.

The ADAM with thrombospondin motifs (ADAMTS) family is a close relative of the ADAM family. ADAMTS members contain a varying number of *C*-terminal thrombospondin type-1 motifs in place of the ADAM transmembrane and cytoplasmic domains and thus function as secreted proteinases [14,15]. Unlike ADAMs, all ADAMTS share the catalytic consensus sequence mentioned above and thus encode active metalloproteinases. The human ADAMTS family includes 19 members that can be sub-grouped on the basis of their known substrates, namely aggrecanases or proteoglycanases (ADAMTS1, 4, 5, 8, 9, 15 and 20), procollagen *N*-propeptidases (ADAMTS2, 3 and 14), cartilage oligomeric matrix protein (also known as thrombospondin-5) cleaving proteinases (ADAMTS7 and 12), von Willebrand factor (VWF) cleaving proteinase (ADAMTS13) and a group of orphan enzymes (ADAMTS6, 10, 16, 17, 18 and 19) [14,15]. The gene name ADAMTS11 was assigned in error to a gene already designated as ADAMTS5, and thus the term ADAMTS11 is no longer used. Mendelian disorders resulting from mutations in ADAMTS2, 10, 13 and 17 identified essential roles for each gene [16]. ADAMTS13 is one of the most studied ADAMTSs because of its critical involvement in a thrombotic disorder [17,18]. ADAMTS13 is the sole VWF-cleaving enzyme in blood plasma and regulates the multimerization state of VWF for proper blood coagulation, and has no other known substrates. A deficiency in plasma ADAMTS13 activity causes thrombotic thrombocytopenic purpura (TTP), a hereditary or acquired (idiopathic) life-threatening disease [17,18,19]. Various lines of evidence indicate that ADAMT4 and 5 are the principal enzymes involved in the degradation of aggrecan, the major proteoglycan in articular cartilage, resulting in the development of osteoarthritis and have thus become targets for therapeutic inhibition [20]. Replacement of the *C*-terminal ancillary domains of ADAMTS5 with those of ADAMTS13 confers the ability to cleave VWF, suggesting that the non-catalytic *C*-terminal domains strongly determine the specificity of ADAMTS5 and ADAMTS13 [21]. The importance of the non-catalytic domains is also supported by the observation that autoantibodies against the ancillary domain of ADAMTS13 can inhibit proteinase activity sufficiently to cause TTP [22]. Different ADAMTS recognize very distinct substrates but the non-catalytic domains that characterize each ADAMTS family member may perform similar functions in other ADAMTS.

Snake venom is a complex mixture of bioactive enzymes and non-enzymatic proteins [23,24]. These toxic compounds appear to have resulted from the convergent or divergent evolution of physiological molecules to have a role in killing and paralyzing prey [25,26]. Snake venom metalloproteinases (SVMPs) have been inferred to be derived through recruitment, duplication and neofunctionalization of ancestral gene encoding closely related ADAM7, 28 and ADAMDEC-1 [27]; therefore, SVMPs are also referred to as snake ADAMs. Actually, large SVMPs, categorized into the P-III class SVMPs, have a modular structure that is homologous to the ectodomain of membrane-anchored ADAMs [28]. SVMPs identified so far share the catalytic consensus sequence and thus are soluble proteinases. Proteomic analyses of snake venoms show that SVMPs constitute more than 30% of the total proteins in many Viperidae venoms and are also present, but are less significant, in the venoms of Elapidae, Atractaspididae and some species of Colubridae [29,30]. These observations suggest that SVMPs play potentially sigc-nificant roles in envenomation-related pathogeneses, such as bleeding, intravascular clotting, edema, inflammation and necrosis [31,32]. SVMPs are the primary factors responsible for local and systemic hemorrhage and may also interfere with the hemostatic system through fibrinogenolytic or fibrinolytic activities, activation of prothrombin or factor *X*, and inhibition of platelet aggregation [33,34,35]. SVMPs are grouped into several classes according to their domain organization (see below). The high molecular weight P-III class SVMPs are characterized by higher hemorrhagic activity than the P-I class of SVMPs, which only have a catalytic metalloproteinase domain. Although SVMP-induced hemorrhages are primarily dependent on SVMP proteolytic activity, the proteolytic activities themselves do not parallel the potency of these activities. The stronger hemorrhagic activity of P-III SVMPs is, at least in part, likely caused by the resistance to inhibition by the plasma proteinase inhibitor α2-macroglobulin (α2M) probably because of the large molecular size of P-III SVMPs: P-I SVMPs are readily inhibited by α2M [36]. P-III SVMPs are capable of inducing not only local but also systemic bleeding, whereas P-I SVMPs mainly induce local hemorrhage [37,38,39]. Therefore, it is more likely that the *C*-terminal non-catalytic domains may contribute to the targeting of P-III SVMPs to relevant molecules in the extracellular matrix of capillaries. P-III SVMPs represent not only higher hemorrhagic activities, but also more diverse and specific biological activities than P-I SVMPs. These observations strongly suggest the importance of the non-catalytic ancillary domains of P-III SVMPs for their functions.

ADAMs, ADAMTSs and SVMPs share a topological similarity with matrix metalloproteinases (MMPs) in the structure of their catalytic domain [40]. However, their non-catalytic ancillary domains are clearly distinct from those of MMPs and other metalloproteinases, thus comprising the M12B clan of zinc metalloendoproteinases (MEROPS classification, https://merops.sanger.ac.uk/). The M12B proteinases are also referred to as adamalysins or repropysins, nomenclatures chosen to reflect the two distinct origins of proteins in this class: the first family member to be structurally characterized was adamalysin II from *rep*tile venom, whereas others belong to a group of proteinases initially described in male *repro*ductive tissues [41,42,43]. Structure–function studies of the M12B proteinases were reviewed several years ago [44,45,46,47]. This review will update our knowledge of the three-dimensional structures of M12B proteinases and describe details of the structural features of their unique domains that may collaboratively participate in directing these proteinases to specific substrates.

## 2. Modular Architecture of ADAMs, ADAMTSs and SVMPs

**Figure 1:**
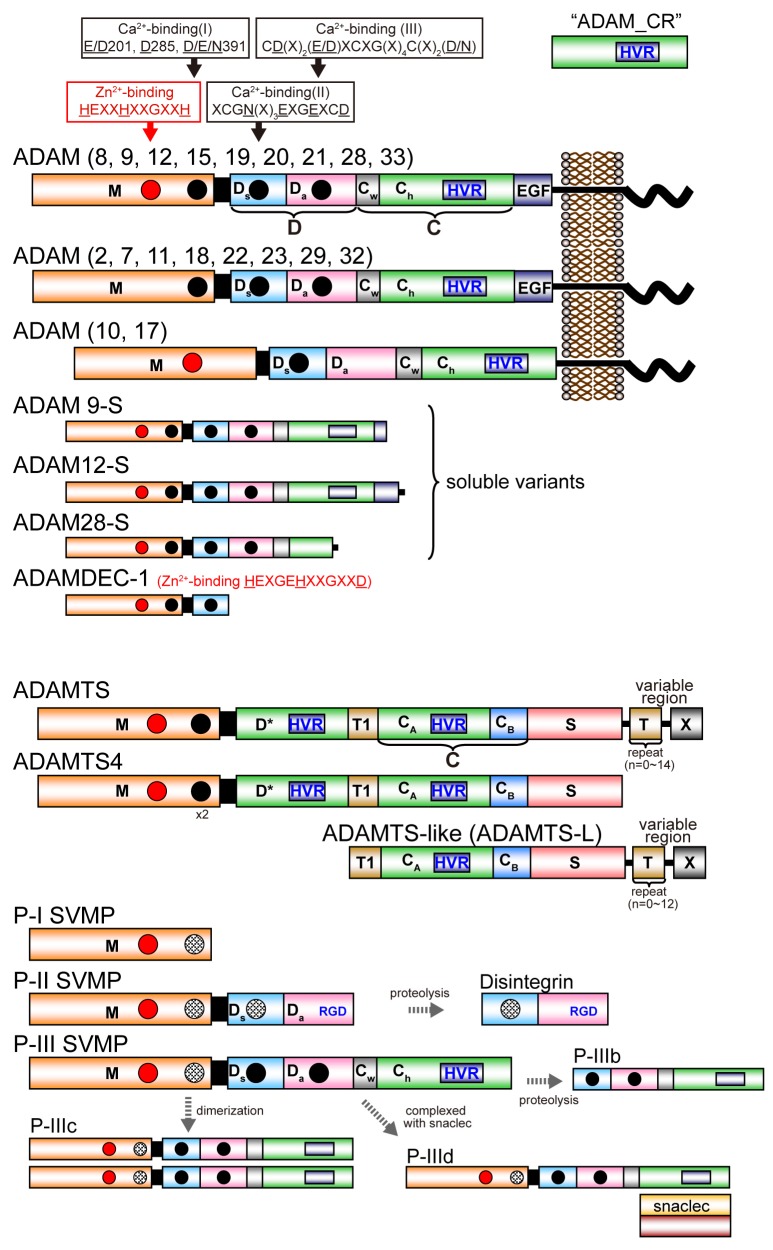
Schematic diagram of the domain structure of M12B proteinases. Each domain or subdomain is represented by a distinct color. The C_h_ subdomain of ADAMs and P-III SVMPs, D* domain of ADAMTSs, and C_A_ subdomain of ADAMTSs and ADAMTS-Ls, adopt the ADAM_CR domain fold and are thus shown in the same color. The region that is variable among ADAMTSs and ADAMTS-Ls is shown as *X*.

Figure 1 depicts the modular domain architectures of M12B clan members. The mature ADAMs generally possess, from *N* to *C* terminus, metalloproteinase (M), disintegrin-like (D), cysteine-rich (C) and epidermal growth factor (EGF) domains, a short connecting linker, a hydrophobic transmembrane (TM) segment and a cytoplasmic tail. ADAM10 and 17 lack an EGF domain and thus, the TM segment follows the MDC domains [28,48]. The D and C domains can be structurally further divided into two subdomains, D_a_ and D_s_, and C_w_ and C_h_, respectively (see below) [28]. The *C*-terminal cytoplasmic tails of ADAMs are very diverse in terms of length (40–250 amino acids) and sequence, and probably do not adopt stable three-dimensional structures. Some ADAMs (ADAM9, 12 and 28) have splicing variants that are expressed as soluble active proteinases without the transmembrane and cytoplasmic regions [49,50,51]. The ADAMDEC-1 (decysin-1) is a unique protein comprising an M domain and a short disintegrin-like domain and is predicted to be secreted as a soluble proteinase [52]. ADAMDEC-1 harbors a putative zinc-binding sequence (HEXXHXXGXXD). However, the third zinc-coordinating residue in ADAMDEC-1 is an Asp instead of the His residues found in all other proteolytically active ADAMs, and thus ADAMDEC-1 is regarded as a member only of a novel subgroup of ADAMs [52].

All mature ADAMTS members commonly possess, from the *N*- to *C*-terminus, metalloproteinase (M), disintegrin-like (D), central thrombospondin type-1 repeat (TSR) motif (T1), cysteine-rich (C) and spacer (S) domains. Despite its name, the D domain of ADAMTSs actually does not adopt a classic “disintegrin-like” tertiary structure, but has an ADAM_CR domain fold (see below) and is thus indicated as “D*” hereafter. The C domain in ADAMTS can be structurally further divided into two distinct subdomains, C_A_ and C_B_ (see below) [53]. ADAMTS4 has this basic core MD*TCS domain organization and other family members have a variety of more distal *C*-terminal domains, including one or more additional TSRs and additional domains denoted as “X” in Figure 1, which are characteristic of particular subgroups [14,15]. In the *C*-terminal region, ADAMTS9 and 20 have a GON-1 domain whereas ADAMTS13 has two CUB (complement components C1rC1s/urinary epidermal growth factor/bone morphogenic protein-1) domains. Several ADAMTSs (ADAMTS2, 3, 6, 7, 10, 12, 14, 16, 17 18 and 19) have a PLAC (protease and lacumin) domain, and ADAMTS7 and 12 have a mucin/proteoglycan domain interposed between TSR4 and TSR5 [14,15]. There are six ADAMTS-like (ADAMTS-L) proteins, which include ADAMT-L1 to 5 and papilin, resemble ADAMTS ancillary domains but lack the M and D domains. ADAMTS-Ls are products of distinct genes, not alternatively spliced variants of ADAMTS genes. ADAMTS-Ls appear to have architectural or regulatory roles in the extracellular matrix instead of a catalytic activity [15]. ADAMTS-L2 is implicated in an inherited connective tissue disorder named geleophysic dysplasia [54]. A homozygous *ADAMTS-L4* mutation was identified in isolated ectopia lentis [55].

SVMPs are classified into three major classes, P-I, P-II and P-III, according to their domain organization [34,56]. P-I SVMPs are composed of a single catalytic M domain. P-II SVMPs are synthesized as an M domain and a D domain. P-III SVMPs have a modular structure homologous to the MDC domains of the membrane-anchored ADAMs. In venoms, P-I and P-III SVMPs are abundant, but P-II SVMPs are frequently found in processed forms containing only their disintegrin domain, *i.e.*, classic disintegrins. P-III SVMPs can be divided further into subclasses depending on their post-translational modifications, such as proteolytic processing between the M and D domains (P-IIIb) or dimerization (P-IIIc), complexation (P-IIId) with additional snake venom C-type lectin-like proteins (snaclecs) [57], in addition to the canonical P-IIIa SVMPs. SVMPs of different classes are often present in the same viper venom. P-III SVMPs are present in the venoms of species of the families Viperidae, Elapidae, Atractaspididae and Colubridae, whereas P-I and P-II SVMPs have been described only in venoms of viperid species [58]. The evolutionary history of viperid SVMPs is characterized by repeated domain loss; the loss of the C domain precedes the formation of the P-II SVMPs, which in turn precedes the evolution of the P-I SVMPs through loss of the D domain [58,59,60].

All M12B proteinase members possess an *N*-terminal signal sequence that directs the proteinase into the secretory pathway. Adjacent to this signal sequence is the pro domain (typically approximately 200 amino acid residues) that has been suggested to assist with the correct folding of the protein and to maintain the proteinase in a latent state via a cysteine-switch [61] or other mechanism [62] until its cleavage either by a pro-protein convertase or by autocatalysis during its transit through the Golgi apparatus. Unlike other M12B members, the pro domain of ADAMTS13 is relatively short (only 41 residues) and is not required for its secretion and function [63].

## 3. Three-Dimensional Structures

The three-dimensional structures currently available for the M12B members are summarized in Table 1. Adamalysin II is a P-I SVMP isolated from *Crotalus adamanteus* and is the first M12B proteinase for which a crystal structure was solved in 1993 [42]. The first mammalian member, the M domain of human ADAM17 (TACE) structure was reported in 1998 [64]. To date, the isolated M domains or M-domain-containing structures of ten P-I SVMPs, seven P-III SVMPs, four ADAMs and three ADAMTSs are available in the Protein Data Bank (PDB). A significant advance in the field was the characterization of the crystal structure of the first P-III SVMP, vascular apoptosis-inducing protein-1 (VAP-1) in 2006 [28]. The structural determination of six P-III SVMPs, including almost all P-III subclasses, followed that of VAP-1. The entire ectodomain structure of mammalian ADAMs is currently only available for ADAM22, which was reported in 2009 [65]. The ADAM22 structure was also the only non-catalytic ADAM for which a crystal structure was solved [65]. Other significant advances are the structural determination of the MD* domains of ADAMTS1 in 2007 [66] and the D*TCS domains of ADAMTS13 in 2009 [53]. The MD*-domain-containing structures of ADAMTS4 and 5 are also available in the PDB. Although no three-dimensional structure of the intact ADAMTS has been determined, a structural model of the core MD*TCS domain of ADAMTS13 has been proposed [53]. No pro domain-containing structures are currently available for M12B proteinases although several zymogen structures of MMPs have been deposited in the PDB [67].

### 3.1. M Domain

**Figure 2:**
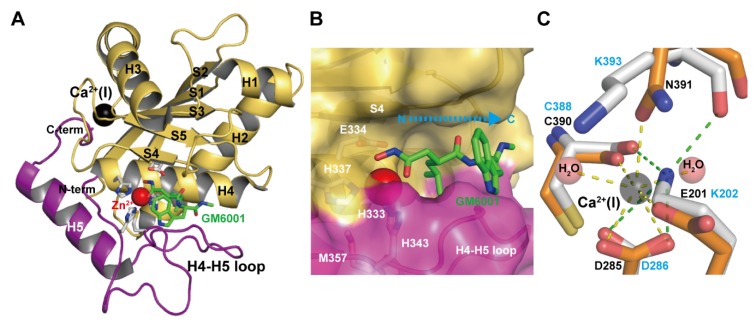
Catalytic M domain structure. (**A**) Structure of the M domain of catrocollastatin/VAP2B in complex with GM6001 (2DW0). The upper and lower subdomains are colored in gold and magenta respectively. (**B**) Close up view of the catalytic site. (**C**) Close up view of the Ca^2+^-binding site of catrocollastatin/VAP2B (shown in orange) overlaid on the corresponding region of VAP1 (shown in gray). Residues in catrocollastatin/VAP2B and VAP1 are indicated in black and cyan, respectively.

The M domains of M12B proteinases range from 180 to 260 (typically 200–210) residues in length [33,87]. The currently available M domain structures of ADAMs, ADAMTSs and all classes of SVMPs are very similar to each other, although comparison of the amino acid sequences of various members shows high variability (typically 20%–50% identity). Interestingly, although the human ADAM8 M domain is most similar in sequence to the human ADAM33 M domain (44% identity), its crystal structure is most similar to that of P-I SVMP adamalysin II [68]. The M domain of the non-catalytic ADAM22 also adopts a very similar backbone structure to those of other catalytic ADAMs, ADAMTs and SVMPs [65]. The M domain of M12B proteinases has a core structure with a conserved molecular topology consisting of a five-stranded β-sheet, four long α-helices, and one short *N*-terminal α-helix. Figure 2A depicts the M domain structure of catrocollastatin/VAP2B, a representative of P-III SVMPs, in complex with the hydroxamic inhibitor GM6001 as viewed from the so-called standard orientation, a frontal view of the horizontally-aligned active site-cleft proposed for the general description of structural features of metalloproteinses [89]. The M domain has an oblate ellipsoidal shape with a notch in its flat side that separates the upper subdomain (about 150 *N*-terminal residues, colored in olive) from an irregularly folded lower subdomain (about 50 *C*-terminal residues, colored in magenta). The active site cleft extends horizontally across the flat surface of the M domain to accommodate the peptidic inhibitor (Figure 2B). The amino acid sequence of the irregular lower domain region is highly divergent among M12B members and is therefore important for substrate recognition because it forms part of the wall of the substrate-binding pocket. Crystal structures of inhibitor-bound M domain complexes suggest that the hydrogen-bond network formed between the extended substrate and the adjacent pocket-flanking regions of the enzyme resembles that of an antiparallel β-sheet, in essence extending the central β-sheet by two strands [46]. The catalytic site is characterized by a consensus HEXXHXXGXXH sequence (residues 333–343 in catrocollastatin/VAP2B sequence), which is conserved not only in M12B members but also across the metzincin superfamily of metalloproteinases, which also contains MMPs, astacins, and serralysins [40,90]. The three conserved histidine residues (His333, His337 and His 343) coordinate the catalytic zinc ion and Glu334 functions as a catalytic base at the bottom of the active site cleft. The conserved Met357, located 12–24 residues downstream of the catalytic consensus sequence, folds into a so-called Met-turn and forms a hydrophobic base beneath the three zinc-binding imidazole rings, a hallmark of the metzincin superfamily of proteinases.

The secondary structure arrangement of the M domain is similar to that of other metzincins, such as astacin and MMPs, except for the large insertion of the H3 helix and the loop between strand S1 and helix H3 [40]. This insertion contributes to the creation of a Ca^2+^-binding site(S), which is unique to M12B proteinases. Most M12B members have one or two (the case for some ADAMTSs) structural calcium ions (Ca^2+^-binding site I) in close proximity to the crossover point of the *N*- and *C*-termini of the M domain opposite the catalytic site (Figure 2C). In catrocollastatin/VAP2B, the Ca^2+^ ion is coordinated by the side-chains of Asp285, Asn391 and Glu201, the main-chain carbonyl oxygen atom of Cys388, and two water molecules in a pentagonal bipyramidal arrangement. Some ADAMs (e.g., human ADAM10 and 17) and SVMPs have substitutions in these Ca^2+^-coordinating residues and thus lack Ca^2+^-binding at this site. For example, Glu201 and Asn391 are replaced by Lys202 and Lys392, respectively, and the distal Nε atom of Lys202 substitutes for the Ca^2+^ ion in VAP1 (Figure 2C) [28]. Replacement of the Ca^2+^-coordinating Glu residue with Lys is also observed in other SVMPs and ADAMs. The high degree of conservation of residues involved in Ca^2+^-binding or in mimicking Ca^2+^-binding might reflect the importance of this region for the structural link between the M and D domains. In addition, Ca^2+^ protects against autoproteolysis at this M/D domain junction [76,91]. In ADAMTS1, 4 and 5, a second bound Ca^2+^ ion is found with a metal-metal distance to the first conserved site of around 4Å. The residues coordinating the second Ca^2+^ ion are not conserved in all ADAMTS sequences, hence the second Ca^2+^ ion at this site may not necessarily be a feature of the ADAMTS family. A distinctive feature of the M domain of the M12B proteinases, when compared to that of MMPs, is the presence of two to four disulfide bonds that stabilize the structure (whereas MMPs have none).

**Figure 3:**
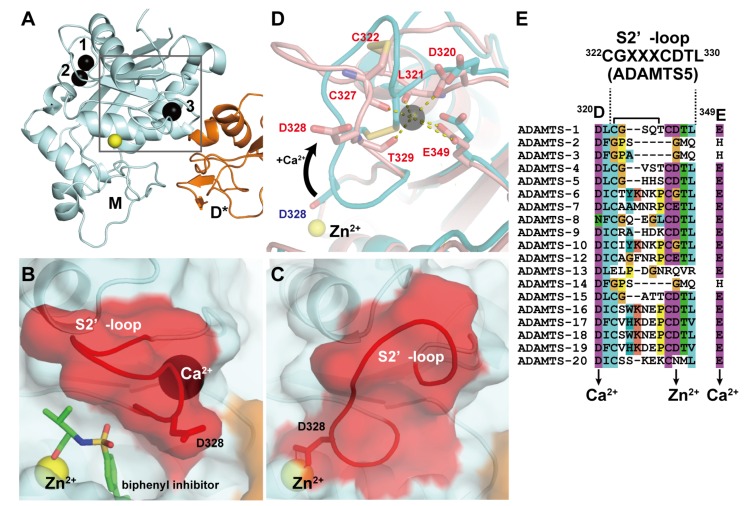
Crystal structures of ADAMTS5-MD*. (**A**) Overall structure of ADAMTS-MD*. Zn^2+^ and Ca^2+^ ions are shown in yellow and black spheres, respectively. Molecular surface of the active site in the inhibitor-bound “open” (**B**) and the apo “closed” form (**C**). (**D**) Superimposition of the two loop configurations: The closed conformation is depicted in cyan, the open conformation is shown in pink. (**E**) Amino acid sequence alignments of ADAMTSs around the “S2’-loop”. The Genbank IDs for each ADAMTS sequence are, ADAMTS1 (Genbank ID (GI): 50845384), ADAMTS2 (GI: 3928000), ADAMTS3 (GI: 21265037), ADAMTS4 (GI: 12643637), ADAMTS5 (GI: 12643903), ADAMTS6 (GI: 64276808), ADAMTS7 (GI: 38197242), ADAMTS8 (GI: 153792351), ADAMTS9 (GI: 33624896), ADAMTS10 (GI: 56121815), ADAMTS12 (GI: 51558724), ADAMTS13 (GI: 21265034), ADAMTS14 (GI: 21265052), ADAMTS15 (GI: 21265058), ADAMTS16 (GI: 32363141), ADAMTS17 (GI: 37999850), ADAMTS18 (GI: 76800647), ADAMTS19 (GI: 29336810) and ADAMTS20 (GI: 28316229).

In ADAMTS4 and 5, in addition to the two Ca^2+^ ions at site I, another bound Ca^2+^ ion has been observed in the M domain in close proximity to the active site (Figure 3A). Crystal structures of the MD* domain-containing fragment of ADAMTS4 in the presence or absence of the inhibitor revealed that the active site of ADAMTS4 adopts two alternative conformations that may exist in equilibrium: an inhibitor-bound “open” structure with an additional Ca^2+^ ion bound (Figure 3B) and an apo “closed” inaccessible structure without a bound Ca^2+^ ion (Figure 3C) [71]. In the open form, the Ca^2+^ ion is coordinated by the side-chain oxygen atoms of Asp320 and Glu349 and the main-chain carbonyl oxygens of Leu321, Cys327 and Thr329. The major difference between these two states is found in the position and conformation of the short disulfide-containing “S2’-loop” encompassing residues 322–330. In the apo state, the S2’-loop moves from its “open” position toward the catalytic Zn^2+^ ion by ~8Å and folds into the active site in a “closed” autoinhibited state in which the side-chain carboxylate of Asp289 chelates the Zn^2+^ ion, resulting in the removal of bound Ca^2+^ ion (Figure 3D). Owing to the strong sequence similarity among ADAMTS4 and other ADAMTSs (ADAMTS1, 5, 8 and 15) in the S2’-loop, which has the consensus CGXXXCDTL sequence, and the Ca^2+^-coordinating Asp320 and Glu349 (Figure 3E), it seems likely that these ADAMTSs may also bind Ca^2+^ and adopt two alternative conformations. ADAMTS13 does not share the S2’-loop sequence with ADAMTS5, but a site-directed mutagenesis study suggested that Ca^2+^-binding to the residues constituting this loop strongly affects the catalytic activity of ADAMTS13 [92]. The crystal structure of the M domain of ADAMTS13 remains to be elucidated. The above consensus sequence and the existence of two distinct conformational states in the M domain have not been observed in either ADAMs or SVMPs.

### 3.2. C-Shaped MDC Domains of ADAMs and P-III SVMPs

**Figure 4:**
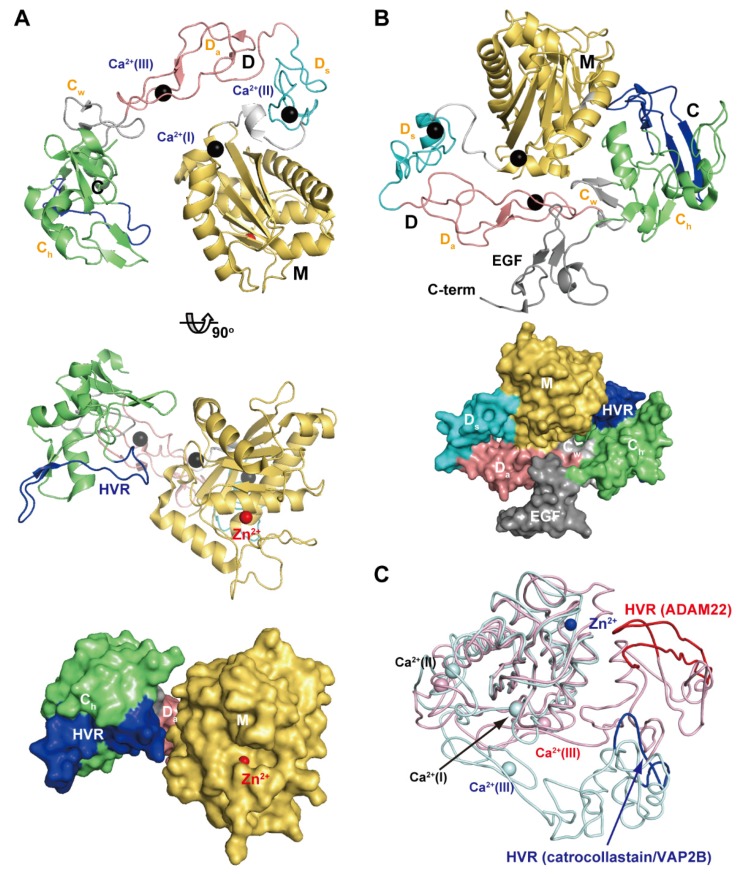
C-shaped MDC-domain configuration of ADAMs and P-III SVMPs. Ribbon and molecular surface representations of the crystal structure of catrocollastatin/VAP2B (**A**) and ADAM22 (**B**). (**C**) Superimposition of the M domains of catrocollastatin/VAP2B (shown in cyan) and ADAM22 (shown in pink).

Figure 4A depicts the crystal structure of catrocollastatin/VAP2B, the first monomeric P-III SVMP structure to be solved [87], representing a structural prototype of P-III SVMPs. The crystal structures of P-III SVMPs reveal that the MDC domains fold into a C-shaped configuration in which the distal HVR portion (see below) of the C domain is situated near to, and faces towards, the catalytic site in the M domain. The complete ectodomain (M/D/C/EGF domains) structure of ADAM22 (Figure 4B) shows that four domains assemble together like a four-leaf clover, each leaf representing one of the four domains [65]. ADAM22 structure reveals that the C-shaped configuration of the MDC domains found in SVMPs are conserved in mammalian ADAMs, and the additional EGF domain is tightly associated with both the D and C domains forming a continuous D/C/E module. In catalytically active ADAMs, the EGF domain may form a rigid spacer that correctly positions the MDC domains against the membrane for the subsequent shedding of membrane-anchored molecules. The D domain is linked to the M domain by a short linker (7–12 amino acid residues) that allows variable orientation and positioning between the M and D domains [28,46,65,87]. Consistent with this, comparison of the available P-III SVMP and ADAM structures reveals substantial diversity in the relative position of the M and D domains [87]. For example, catrocollastatin/VAP2B shows an open C-shaped molecule with no direct interaction between the M and D domains except at the domain junction, whereas the two domains directly interact with each other in ADAM22 and thus adopt a closed C-shaped structure (Figure 4C). The flexibility of the molecule is reflected in the ability of the same proteins to crystallize in different crystal forms, and *vice versa* [93]. The structures of ADAMs and P-III SVMPs are most likely dynamic, allowing for a varying distance between the M domain and the rest of the molecule. This intrinsic flexibility may be important for fine-tuning substrate recognition, by adjusting the spatial alignment between the catalytic region and the exosite (see below) during the catalytic cycle.

In some instances, substantial amounts of processed DC fragments of P-IIIb SVMPs have been identified in venoms alongside their unprocessed counterparts [94,95]. Although lacking proteolytic activity, such isolated DC fragments display diverse biological activities, such as inhibition of collagen-stimulated platelet aggregation and the modulation of cell adhesion, migration, and proliferation, implying that the DC fragments derived from P-IIIb SVMPs are also important in the toxicity of the venoms [33,56]. Some membrane-anchored ADAMs, such as ADAM2 (fertilin-β) and ADAM1 (fertilin-α), undergo proteolytic processing within the M/D-linker and the Ca^2+^-binding site III (see below), respectively, at different stages of sperm maturation [12,96]. A flexible modular structure, in addition to Ca^2+^-binding, may also play a role in differential proteolytic processing of precursor proteins, giving rise to the functional complexity of snake venoms, as well as in the post-translational regulation of ADAMs’ functions, probably by modifying the capabilities of protein–protein interactions.

### 3.3. Arm Structure in ADAMs and P-III SVMPs

**Figure 5:**
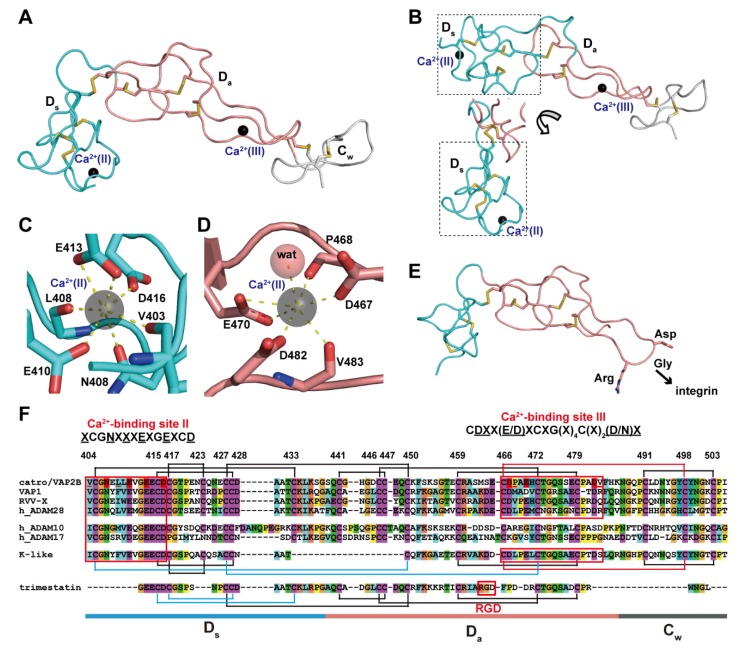
Arm structure. (**A**) The D_s_, D_a_, and C_w_ subdomains of catrocollastatin/VAP2B (2DW0) are shown in cyan, pink, and gray, respectively. (**B**) The D domain of K-like proteinase (3K7N) with two different views of the D_s_ subdomain (in dotted line boxes). Close up views of the Ca^2+^-binding sites, II (**C**) and III (**D**) in catrocollastatin/VAP2B. (**E**) Structure of an RGD-containing disintegrin, trimestatin (1J2L). Suggested integrin-binding residues are indicated. (**F**) Amino acid sequence alignment of catrocollastatin/VAP2B (PDB: 2DW0_A), VAP1 (PDB: 2ERO_A), RVV-X (PDB: 2E3X_A), human ADAM28 (Genbank ID (GI): 98985828), human ADAM10 (GI: 29337031), human ADAM17 (GI: 14423632), K-like (PDB: 3K7Y_A) and trimestatin (1J2L_A) generated using Clustal *X*2 (http://www.clustal.org/clustal2/). Disulfide bonds and the boundaries of the subdomains are schematically indicated. Ca^2+^-binding sites II and III are boxed in red.

The D domain that follows the M domain of ADAMs and P-III SVMPs can be further subdivided into two structural subdomains, the “shoulder“ (D_s_, residues 403–436 in catrocollastatin/VAP2B sequence) and the “arm“ (D_a_, residues 437–486) [28] (Figure 5). Both subdomains consist largely of a series of turns and constitute an elongated curved arm structure together with the immediately subsequent region of the primary sequence, the *N-*terminal region of the C domain designated as the “wrist“ (C_w_, residues 437–503) subdomain (Figure 5A). The structure of the entire C-shaped arm (D_s_/D_a_/C_w_) itself seems to be rigid because it is stabilized by a number of disulfide bonds and structural Ca^2+^ ions. There are three disulfide bonds in each D_s_ and D_a_, and one in C_w_, with the subdomains (e.g., D_s_/D_a_ and D_a_/C_w_) connected by single additional disulfide bonds. The numbers and spacing of the cysteine residues involved in these disulfide bonds are strictly conserved among ADAMs and P-III SVMPs [28,87] (Figure 5F), with few exceptions, one of which is the kaouthiagin-like (K-like) SVMP from *Naja atra*. The K-like proteinase lacks the 17-amino acid segment at the junction of the D_s_ and D_a_ subdomains, resulting in a different disulfide-bond pattern in the D domain. Consequently, the K-like proteinase has a different orientation between the D_s_ and D_a_ subdomains when compared to that of catrocollastatin/VAP2B (Figure 5B), and thus the MDC domains of K-like proteinase adopt a more elongated, I-shaped configuration [85]. However, how this I-shaped structure correlates with the proteinase function remains to be elucidated.

Both the D_s_ and D_a_ subdomains contain structural Ca^2+^-binding sites that were not predicted from the amino acid sequences [28,87]. In the D_s_ subdomain, the side-chain oxygen atoms of the highly conserved Asn408, Glu412, Glu415 and Asp418 (represented by the consensus sequence *X*CGN(*X*)_3_E*X*GE*X*CD, in which the side-chains of underlined residues are involved in Ca^2+^-binding) and the main-chain carbonyl oxygen atoms of Val405 and Phe410 are involved in pentagonal bipyramid coordination of the Ca^2+^-binding site II (Figure 5C). On the other hand, the side-chain oxygen atoms of Asp469, Asp472 and Asp483 and the main-chain carbonyl oxygen atoms Met470 and Arg484, as well as a water molecule, coordinate the Ca^2+^ ion at the corner of a pentagonal bipyramid and constitute the Ca^2+^-binding site III in the D_a_ subdomain (Figure 5D). These residues are also highly conserved among all known ADAMs and P-III SVMPs, with the exception of ADAM10 and 17, and are represented by the consensus sequence CD(*X*)_2_(E/D)*X*C*X*G(*X*)_4_C(*X*)_2_(D/N) [28,87]. Both bound Ca^2+^ ions in sites II and III are deeply buried and tightly coordinated and cannot be stripped from ADAM22, even using EDTA [65]. Therefore, these Ca^2+^ ions are likely to remain permanently in place once the D domain is folded.

The overall structures of the D domain of P-III SVMPs and ADAM22 are similar to that of trimestatin, an RGD (Arg-Gly-Asp sequence)-containing classic disintegrin [97] (Figure 5E). The integrin-binding ability of disintegrins has been attributed to a highly mobile hairpin loop (disintegrin loop) that contains the cell-adhesion sequence RGD at its tip. In ADAMs and P-III SVMPs, the RGD sequence is usually replaced by an (D/S)XCD sequence (residues 466–469 in the catrocollastatin/VAP2B sequence). The disintegrin-like loops of P-III SVMPs and ADAMs are packed against the subsequent C_w_ subdomain, and a disulfide bond (Cys468/Cys499) and bound Ca^2+^ ion at site III further stabilize the continuous rigid D_a_/C_w_ structure. Therefore, in ADAMs and P-III SVMPs, the disintegrin-like loop is inaccessible for protein–protein interactions due to steric hindrance. Disintegrins (40–100 amino acids) are typically generated by proteolytic processing of larger precursor P-II SVMPs [98,99,100], albeit with some exceptions [101]. Most P-II SVMPs have two to four fewer cysteine residues in the D_s_ subdomain than P-III SVMPs, and thus one or two fewer disulfide bonds. In addition, there are substitutions of the key residues constituting the Ca^2+^-binding site II and III in most P-II SVMPs [87]. Although a number of disintegrin structures have been determined by NMR and *X*-ray crystallography [100], no structural Ca^2+^-binding has been identified in these structures and the D_s_ subdomain region of disintegrins is generally shorter and less ordered than the corresponding regions of ADAMs and P-III SVMPs. Because of the lack of structural Ca^2+^ ions, disintegrin structures are more flexible throughout the molecule, than the corresponding region of ADAMs and P-III SVMPs. The flexibility of RGD-containing disintegrin loops is probably important for the binding of integrins. As previously mentioned, P-II SVMPs may have evolved from ancestral P-III SVMP genes after losing the genetic information encoding the protein regions downstream of the D domain [58,59,60]. Removal of structural constraints (disulfide bonds and structural Ca^2+^-binding sites), imposed both on the disintegrin loop and the D_s_ subdomain in the ancestral P-II SVMPs, has been postulated as the key event that permitted the subsequent evolution of both integrin-binding activity and the proteolytic release mechanism.

While the pattern of disulfide-bond pairing in the D domain determined thus far is strictly conserved among ADAMs and P-III SVMPs, with the exception of K-like proteinase, it may be possible that multiple structural isoforms of the same SVMPs exist in the venom, perhaps as the result of alternative disulfide-bond pairing [102]. For example, the disintegrin bitistatin, which is derived from the precursor P-II SVMP, adopts at least two distinct conformations, the result of different disulfide-bonding patterns [103]. Recently, protein-disulfide isomerase (PDI) was implicated in the regulation of shedding activity of ADAM17 [104], and an NMR structural analysis of the C_h_ subdomain of ADAM17 revealed that PDI can act on this subdomain and convert it from the inactive to the active conformation by disulfide-bond isomerization [69].

### 3.4. ADAM_CR Domain, Another Hallmark of M12B Proteinases

The C domain of ADAMs and P-III SVMPs, typically about 80–150 amino acid residues, can be structurally subdivided into the “wrist” (C_w_, residues 437–503) and the “hand“ (C_h_, residues 504–609 in catrocollastatin/VAP2B sequence) subdomains [28,87]. As mentioned, the C_w_ subdomain tightly associates with the D domain, and the two are integrated into one continuous structure. On the other hand, the C_h_ subdomain constitutes a separate unit and has a unique structure consisting of irregularly folded loops with a core α/β-fold and four to five disulfide bonds. The C_h_ subdomain has a novel fold with no structural similarity to any currently known proteins, with the exception of the corresponding segments of M12B proteinases. The whole C domain of P-III SVMPs and ADAMs has been deposited in the Conserved Domain Database (CDD, http://www.ncbi.nlm.nih.gov/cdd) and the Pfam database (http://pfam.xfam.org/) as the ADAM_CR domain (cl15456 and PF08516, respectively). Here, we define the C_h_ subdomain of ADAMs and SVMPs and corresponding regions of ADAMTSs (D* domain and C_A_ subdomain, see below) as the ADAM_CR domain in a more restricted sense.

Crystallographic studies on the D* domain-containing fragments of ADAMTS1, 4, 5 and 13 revealed that the D* domain of ADAMTSs has no structural similarity to classic snake disintegrins, but is very similar in structure to the C_h_ subdomain of ADAMs and P-III SVMPs [44,53,66,71]. The *N*-terminal portion of the C domain of ADAMTSs (the C_A_ subdomain) also possesses essentially the same fold as the C_h_ subdomain, even though the two share no apparent sequence similarity [53]. Thus while the “disintegrin” nomenclature has been used to describe ADAMTS family proteinases, ADAMTSs actually contain no disintegrin-like structures, but instead have two homologous domains that belongs to the ADAM_CR. Therefore, it is now obvious that the presence of the evolutionarily-conserved ADAM_CR domain, not the disintegrin domain, is another hallmark of the M12B members in addition to the catalytic M domain architecture.

**Figure 6:**
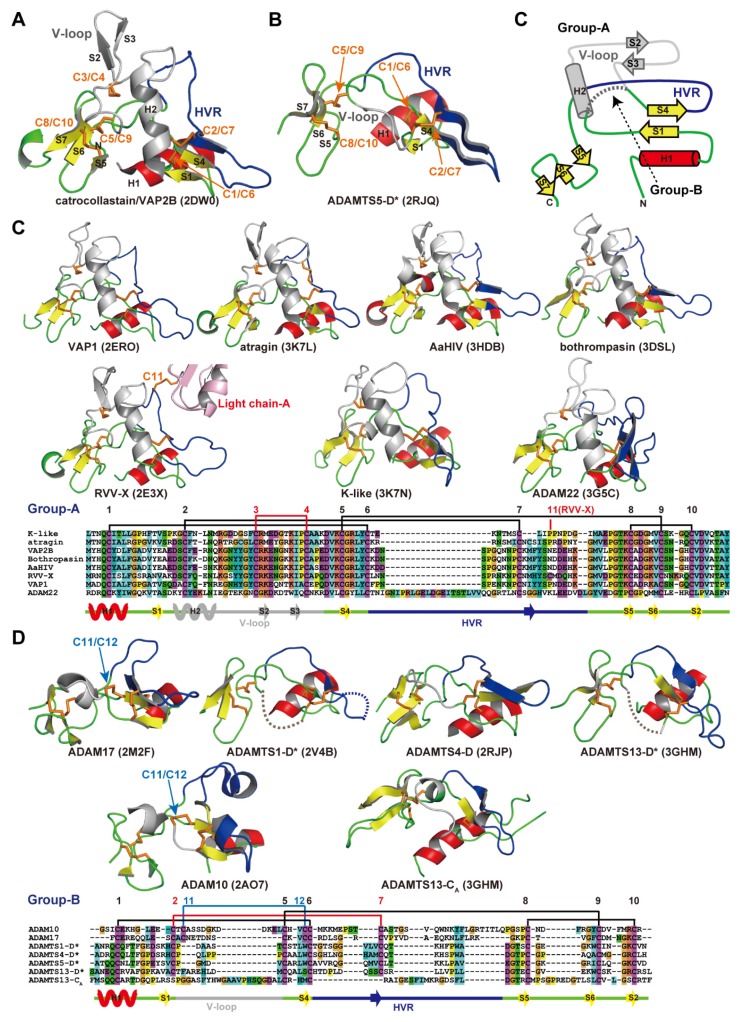
ADAM_CR domain. Ribbon representation of the C_h_ subdomains of catrocollastatin/VAP2B (**A**) and the D* domain of ADAMTS5 (**B**). (**C**) Topology diagram of the ADAM_CR domain. Gallery of the group-A (**C**) and group-B (**D**) ADAM_CR domain structures. Conserved α-helix and β-strands are shown in red and yellow, respectively. Disulfide bonds, residues in HVR, and the residues in the V-loop are shown in orange, blue, and gray, respectively. The PDB ID for each protein structure is indicated in parentheses.

Figure 6A,B depict ribbon representations of the C_h_ subdomain of catrocollastatin/VAP2B and the D* domain of ADAMTS5, respectively, two typical ADAM_CR domain structures. Although there is negligible sequence identity between these two protein portions (~16%), they clearly show similar topologies. The topology diagram of these two protein portions is shown in Figure 6C. The conserved regions are a core α-helix (shown in red), two sets of short β-sheets (shown in yellow), and four disulfide bonds (shown in orange). Major differences between the two molecules are observed in the segment between the two *N-*terminal strands, S1 and S4, shown in gray. A short connecting loop of six amino acids in ADAMTS5 is replaced by a 27 amino acid residue insertion forming a central α-helix and two consecutive hairpin loops protruding out the top of the molecule in the case of catrocollastatin/VAP2B. This segment is named variable loop (V-loop) [28,53]. Current ADAM_CR domain structures can be classified into two groups according to the length of their V-loop. All of the C_h_ subdomains of SVMPs determined thus far and ADAM22 show a catrocollastatin/VAP2B type long V-loop structure (classified as group-A, Figure 6C), whereas ADAM10 and 17, and the D* domains and C_A_ subdomains of ADAMTSs have a short ADAMTS5-D* type V-loop (classified as group-B, Figure 6D). Inspection of the amino acid sequence alignments of other M12B members suggests that the C_h_ subdomains of all known P-III SVMPs and ADAMs, except for ADAM10 and 17, are classified into group-A, whereas the D* and C_A_ domains of ADAMTSs are classified into group-B. The V-loop exhibits a high level of variability among the group-B ADAM_CR structures (Figure 6D), comparable to that of the HVR (see below), while the structure of the V-loop in group-B molecules in general is quite mobile and potentially functions as a protein-protein interaction site in addition to the HVR (see below).

The overall structure of the C_h_ subdomain of catrocollastatin/VAP2B is very similar to that of six other SVMPs and that of ADAM22, with variability occurring mostly in loop regions. Of note, aside from the V-loop, the loop encompassing residues 561–582 (catrocollastatin/VAP2B sequence, shown in blue in Figure 6A,C) and extending across the central region of the C_h_ subdomain is the most variable both in length (16–22 amino acids in SVMPs and 27–55 amino acids in human ADAMs) and in amino acid composition. Therefore, this region has been designated as the hypervariable region (HVR) [28,44]. The HVRs in ADAMTSs are relatively short (13–17), but also show variability in their amino acid sequences when compared with different ADAMTSs and ADAMTS-Ls [53]. In ADAM22 and SVMP structures, the HVR is present at the distal end of the C-shaped MDC domains, and points toward and is situated close to the catalytic site of the M domain (Figure 4). This raises the intriguing possibility that the HVR creates an exosite for substrate binding [28,44]. Different ADAMs and SVMPs have distinct HVR sequences, resulting in distinct molecular surface features. Therefore, in addition to the V-loop, the HVR might have a role in specific protein-protein interactions for the cleavage by the M domain, providing a structural correlate for the diversity of biological activities characteristic of ADAMs and P-III SVMPs. The D domain is located opposite to and apart from the M domain active site and thus plays a primary role as a scaffold that spatially allocates two functional units, the catalytic site and exosite, to both ends of the C-shaped molecule.

Several reports suggest that the HVR region directly contributes to the substrate recognition of ADAMs and SVMPs. Most of these studies, however, used synthetic peptides derived from the HVR region or the isolated domains expressed in *E. coli* for functional assays. It should be noted that short peptides or *E. coli* expressed cysteine-rich proteins do not always mimic their counterparts in the intact molecule. The whole C domain or DC domains of ADAMs are suggested to be involved in protein–protein interactions [105,106,107,108]. The acidic surface pocket, which is located apart from both HVR and the V-loop within the C domain of ADAM10, defines cleavage specificity in Eph/ephrin signaling [48]. Recently, the membrane proximal domain (MPD, corresponding to the C_h_ subdomain in this text) of ADAM17, was shown to be responsible for recognition of two type-I transmembrane substrates, the IL-6R and the IL-1RII, but not for the interaction with the type-II transmembrane molecule TNF-α [109]. Further studies identified that the membrane proximal amphipathic 17 amino acid segment, which has the ability to bind lipid bilayers *in vitro*, is also involved both in substrate recognition and in regulating the shedding activity of ADAM17 [110,111], as well as MPD, which functions as a PDI-dependent molecular switch [69]. Most of these studies, however, do not identify specific regions of the C domain involved in the interactions, and the molecular mechanisms underlying substrate recognition remain to be elucidated. There are no systematic structure-based mutagenesis studies of the HVR region or the V-loop of particular ADAMs or SVMPs, and thus there is still no clear evidence establishing that these regions actually form an exosite. In contrast to the situation for ADAMs and SVMPs, the HVR and the V-loop in the D* and the C_A_ domains of ADAMTS13 have actually been shown to constitute VWF-binding exosites (see below).

### 3.5. Structures of Subclasses of P-III SVMP

Proteins with multimer and/or heterogeneous complex structures are frequently observed in snake venoms. Such multimers or protein complexes generally exhibit markedly enhanced pharmacological activities compared to the individual components and thus may play significant roles in snake venom toxicity [112]. Some SVMPs exist as a homo- or hetero dimer (P-IIIc) or as a hetero trimer (P-IIId). The formation of dimers or higher-order oligomers is not uncommon within M12B members. ADAMTS5 can form oligomers and this oligomerization is required for full aggrecanase activity [113]. Early purifications of ADAMTS2 and 13 indicated that these enzymes formed oligomers [114,115], however, there has been no further characterization of these oligomers. Membrane-bound ADAMs, ADAM17 [116] and the sperm-specific ADAMs, such as ADAM2 and 3 [12], exist as multimers in the cell membrane. However, how the multimeric state of these ADAMs and ADAMTSs relates to their functions is largely unknown.

**Figure 7:**
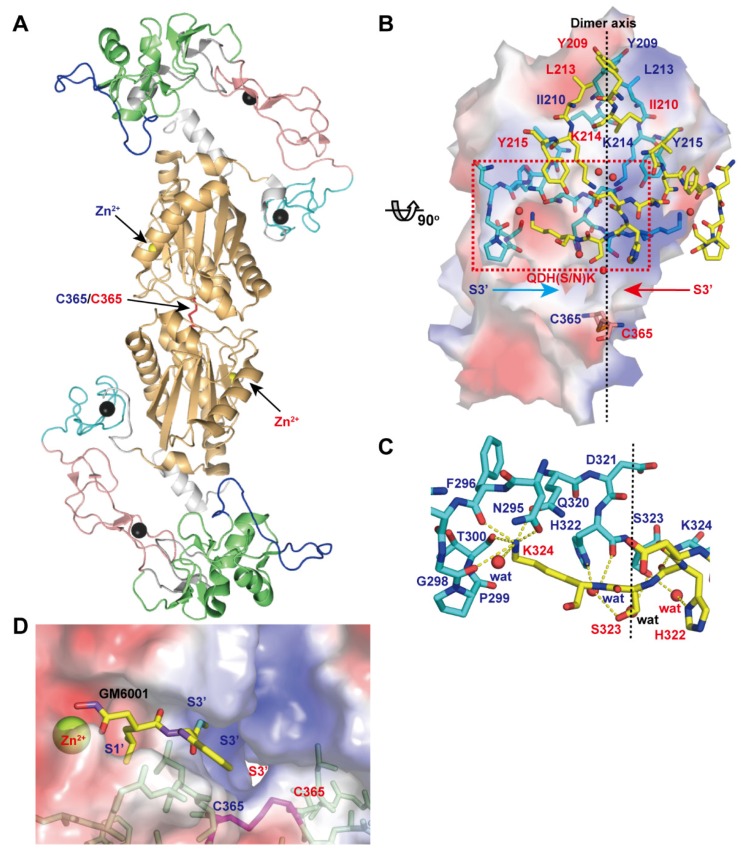
Structure of VAP1, representative of P-IIIc SVMPs. (**A**) Crystal structure of VAP1 (2ERO) viewed from the dimer axis. (**B**) Dimer interface viewed from a direction nearly perpendicular to the dimer axis. The molecular surface of the cyan molecule in the back is colored according to electrochemical potential (red to blue). (**C**) Close up view of the dimer interface. The residues involved in the inter-chain interactions are indicated with blue and red letters for cyan and yellow molecules. (**D**) Close up view of the catalytic cleft of the VAP1 (shown with the molecular surface)/GM6001 (shown in yellow) complex structure.

Figure 7A depicts the crystal structure of VAP1, a homodimeric P-IIIc SVMP. The structure revealed an inter-chain disulfide bond formed between symmetry-related Cys365 residues and some features that characterize P-IIIc SVMPs [28]. The top of the dimer interface is capped by hydrophobic interactions involving Tyr209, Ile210, Leu213, and Tyr215 and the aliphatic portion of Lys214 (Figure 7B). At the middle, there are specific interactions that are best characterized by the QDHSK sequence (residues 320–324 in VAP1) (Figure 7C). The *C*-terminal region of this segment (residues 322–324) forms an antiparallel β-sheet with its counterpart. In addition, water molecules are bound to the side-chain oxygen atoms of His322 and Ser323 and form a hydrogen-bond network that further stabilizes the interface between the monomers. Lys324 plays a pivotal role in the key-to-keyhole recognition between the monomers. The Nε amino group of Lys324 is coordinated by six oxygen atoms, which belong to the opposite chain and are located at the corners of a pentagonal pyramid. The six atoms include the side-chain oxygen atoms of Asn295 and Gln320, the carbonyl oxygen atoms of Phe296, Gly298 and Thr300, and a water molecule (Figure 7C). The intermolecular disulfide bond, located at the bottom of the dimer interface, and the residues in the QDHSK sequence constitute the wall of the substrate-binding S3’ pocket which merges with its counterpart inside the molecule (Figure 7D). Therefore, the two catalytic sites in the dimer are located back-to-back and share their S3’ pockets, suggesting that the two catalytic sites in P-IIIc SVMPs may work in a cooperative manner. VAP1 induces cell death in vascular endothelial cells in culture with all the characteristic features of apoptosis [117]. However, the physiological target(s) of VAP1, the underlying mechanism of VAP1-induced apoptosis, and how dimerization relates to the substrate preference and/or activity of VAP1 remain totally unknown. In addition to VAP-1, HV1 (Genbank ID (GI): 14325767), halysase (GI: 60729695), VLAIP (GI: 82228618), TSV-DM (UniProt ID: Q2LD49.1) and VaH3 (GI: 496537199) are reported to exist in their native states as homo- or heterodimers. In addition to these SVMPs, agkihagin (Uniprot ID: Q1PS45) and halysetin (Uniprot ID: Q90Y44) also share Cys365 and the QDH(S/N)K sequence and thus, these SVMPs can be considered to be P-IIIc SVMPs. Bilitoxin-1 (GI: 172044534) [118], a unique homodimeric P-II SVMP, has neither a cysteine residue at position 365 nor the QDH(S/N)K sequence, suggesting that its dimer interface is different from that of VAP1. Cys365 and the QDH(S/N)K sequence are not found in either ADAMs or ADAMTSs.

A few P-III SVMP members exist as heterocomplexes due to the existence of an extra subunit that interacts through covalent or non-covalent interactions. The venom of Russell’s viper (*Daboia russelli*) has been recognized for its potent coagulation activity. Two major components, RVV-X and RVV-V, of this venom can collaboratively accelerate formation of the prothrombinase complex (Factor *X*a (FXa)/Factor Va (FVa) complex) that converts prothrombin to thrombin, resulting in a disseminated intravascular coagulation in the body of the prey [119]. RVV-X is a unique high molecular weight metalloproteinase, a representative of P-IIId SVMPs. RVV-X activates factor *X* (FX) by cleaving the Arg194-Ile195 bond in FX, which is also cleaved by factors IXa and VIIa during physiological coagulation [120,121]. Because of its extremely high specificity for FX, RVV-X is widely used in coagulation research and in diagnostic applications. A similar FX-activating P-IIId SVMP, VLFXA, has also been isolated from *Vipera lebetina* venom [121,122]. On the other hand, another component RVV-V is a thrombin-like serine proteinase that specifically activates factor V (FV) [123,124].

**Figure 8:**
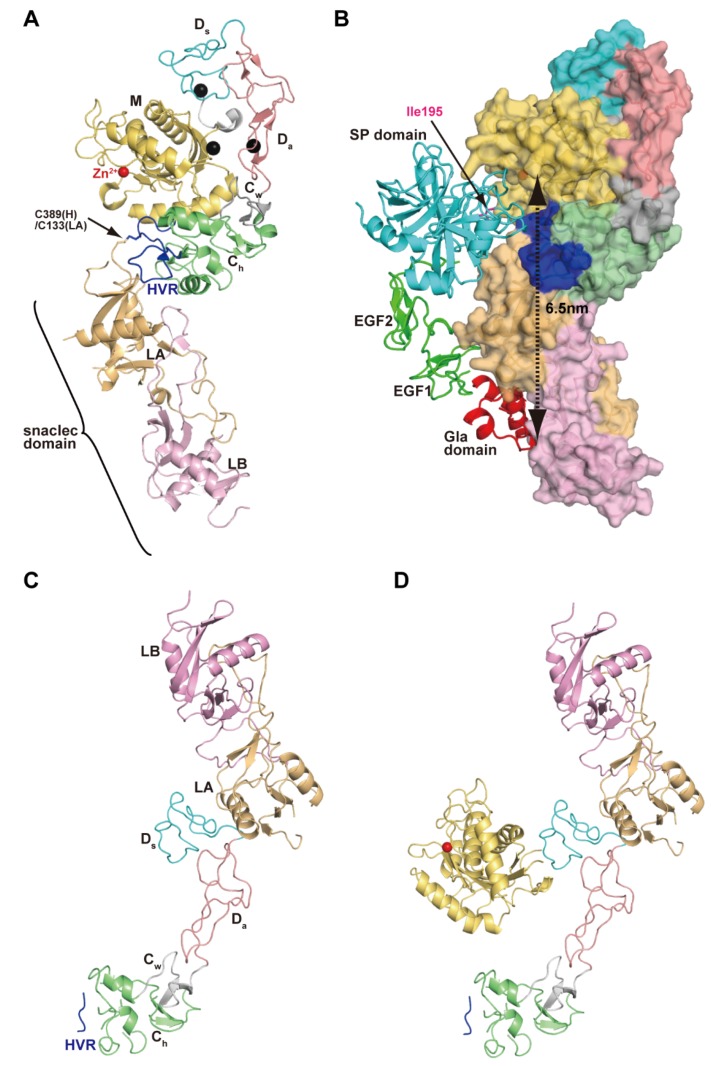
Structures of RVV-X and multactivase, two representatives of P-IIId SVMPs. (**A**) Ribbon representation of the crystal structure of RVV-X. (**B**) Factor *X*a docking model. (**C**) Ribbon representation of the crystal structure of multactivase-ΔM. (**D**) A model of the whole multactivase molecule. The model was constructed by a superimposition of the crystal structures of multactivase-ΔM and of the M/D_s_ domains of catrocollastatin/VAP2B (2DW0). Each subdomain is in a different color.

RVV-X is a heterotrimeric complex consisting of an MDC-containing heavy and two light chains [120,125]. Two light chains form a domain-swapped dimer [126] with features characteristic of snake venom C-type lectins (snaclecs [57]). Instead of binding to carbohydrate moieties, snaclecs bind to membrane receptors, coagulation factors and other proteins essential for hemostasis. The crystal structure of RVV-X revealed its unique hook-spanner-wrench configuration (Figure 8A), in which the MD domains constitute the hook, and the remainder of the molecule forms the handle [88,127]. The backbone structure of the heavy chain is essentially the same as those of other P-III SVMPs. RVV-X has a unique cysteine residue (Cys389), not found in other classes of SVMPs, in the middle of the HVR in the C_h_ subdomain. Cys389 forms a disulfide bond with the *C*-terminal cysteine residue (Cys133) of the light chain-A (LA). In addition, the residues in the HVR and the surrounding regions in the heavy chain form multiple aromatic and hydrophobic interactions and hydrogen bonds with the *N*- and *C*-terminal residues in LA, further stabilizing the continuous C/LA structure. The RVV-X structure provides the first direct observation of a protein–protein interaction mediated by HVR.

The structure of the snaclec domain of RVV-X is quite similar to that of the FX-binding protein (X-Bp) whose crystal structure was solved in complex with the γ-carboxyglutamic acid (Gla) domain of FX [128]. This structural similarity, along with the surface chemical properties and previous biochemical observations, suggests a docking model for FX (Figure 8B) [88,127]. The snaclec domain forms a Gla-domain-binding exosite that may serve as the Ca^2+^-dependent primary capture site for circulating FX. The docking model indicates that the C_h_/snaclec domains act as a scaffold to accommodate the elongated FX model. The relatively large separation (~65 Å) between the catalytic site and the exosite explains the high specificity of RVV-X for FX. This is in sharp contrast to thrombin-like RVV-V which cleaves the Arg1545-Ser1546 bond specifically by recognizing the side-chains of Ile1539 (P7)-Arg1545 (P1) located in close proximity to the scissile bond of FV [129]. The RVV-X structure represents a good example of the evolutionary acquisition of ligand-binding specificity by ADAMs and SVMPs.

Carinactivase-1 and multactivase are potent prothrombin activators isolated from the venom of *Echis carinatus* and *Echis multisquamatus*, respectively [130,131]. They have a snaclec domain in addition to MDC domains, and also use their snaclec domain for prothrombin recognition. Therefore, they are considered to be another example of P-IIId SVMPs. Unlike RVV-X, these two P-IIId SVMPs do not possess a disulfide bond between the heavy chain and snaclec domains, and thus how the catalytic and the regulatory domains interact and are oriented with respect to each other remains unclear. A crystal structure of the proteolytic fragment of multactivase, named multactivase-ΔM because it lacks the M domain from the intact molecule, was recently determined at 2.6Å resolution (Figure 8C) and a structural model of the entire multactivase molecule (Figure 8D) was constructed (*S. Takeda* and *T. Morita*, unpublished work). Each subdomain in multactivase is similar in structure to the corresponding one in RVV-X. However, the interactions between the heavy chain and the snaclec domain are remarkably different. The snaclec domain interacts with the D_s_ subdomain in multactivase but the C_h_ subdomain in RVV-X, resulting in a different overall shape and configuration of the catalytic site and the exosite between these two P-IIId SVMPs. The multactivase structure represents the first crystallographic observation of the interaction between an ADAM D domain and another polypeptide chain, providing additional insights into protein–protein interactions by the M12B clan of proteinases.

### 3.6. Core Structure of ADAMTSs

**Figure 9:**
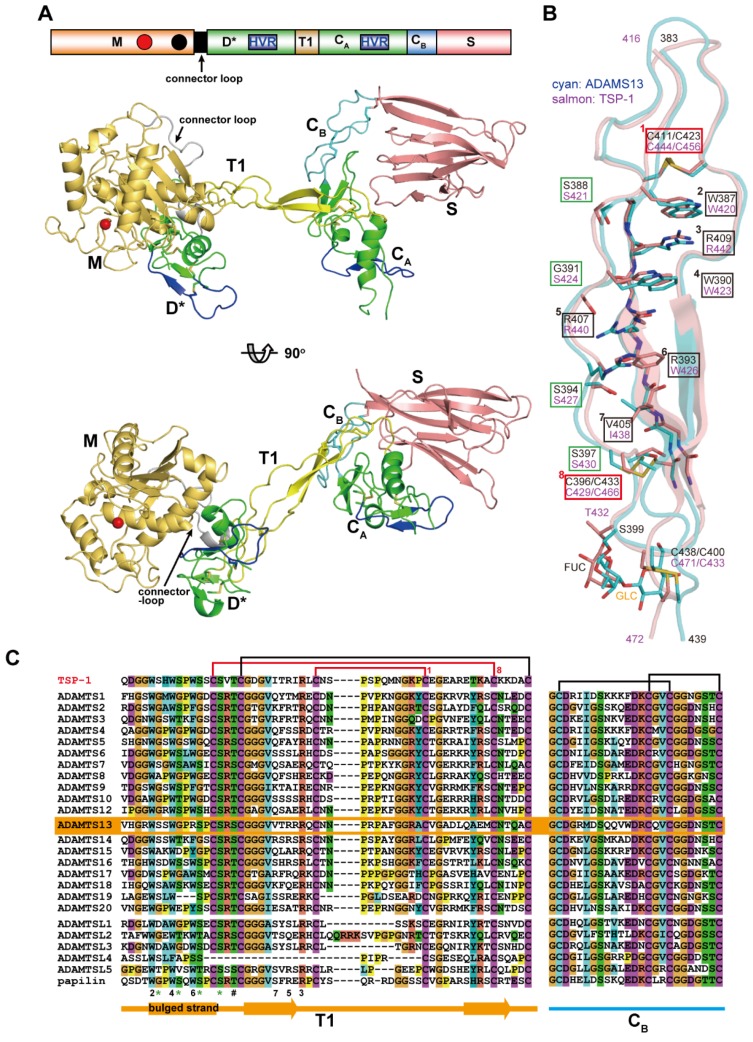
Structure of the core MD*TCS domains of ADAMTS proteinases. (**A**) A structural model of the MD*TCS domains of ADAMTS13 in two different views. (**B**) T1 structure of ADAMTS13 (shown in cyan) superposed onto the TSR2 in TSP-1 (PDB ID: 1LSL, shown in salmon). The residues that form the CWR-layered core (boxed in black and red), the serine residues in the bulged strand that form the hydrogen bond network (boxed in green) and the *O*-linked carbohydrate are indicated. (**C**) Sequence alignment of the T1 and the C_B_ subdomain regions of human TSP-1, ADAMTSs, ADAMTS-L and papilin. The residues involved in the CWR-layered core are indicated by layer number. Conserved serine residues and *O*–linked glycosylation sites are marked with * and #, respectively. The GI numbers for each ADAMTS-L sequence are, ADAMTS-L1 (GI: 37181773), ADAMTS-L2 (GI: 1232266328), ADAMTS-L3 (GI: 145275198), ADAMTS-L4 (GI: 187954849), ADAMTS-L5 (GI: 115311311) and papilin (GI: 145309328).

Figure 9A depicts a structural model of the MD*TCS domains of ADAMTS13 constructed based on the crystal structures of the MD* domains of ADAMTS5 [71] and the D*TCS domains of ADAMTS13 [53]. This model represents the basic architecture of the core portion commonly found in ADAMTS family proteinases. The structure of the core MD*TCS domains consists of three globular knobs, corresponding to the MD*, C_A_ and S domains, which are connected by two elongated structural modules, T1 and C_B_. Unlike ADAMs, ADAMTSs lack the D_s_/D_a_/C_w_ arm structure, and the D* domain with an ADAM_CR domain fold is directly connected to the M domain by a connector loop (16–20 residues) that wraps around the opposite surface of the catalytic site [66,71]. The D* domain stacks against the M domain active site cleft, forming a continuous MD* unit, and potentially provides an auxiliary substrate-binding surface (see below). The side-chain of Phe216 in the M domain points toward, and makes a number of van der Waals contacts with, the small hydrophobic pocket formed in the D domain, thus playing a pivotal role in the interaction between the M and D domains. The F216E mutant, designed to impair the interactions between the M and D* domains, completely lost catalytic activity for the synthetic ADAMTS13 substrate FRET-VWF73 [132] although the secretion level was not greatly reduced [53]. On the other hand, the mutant that increased the stability of the association between the M and D* domains due to the introduction of an extra disulfide bond between the two domains, retained a catalytic activity indistinguishable from that of wild-type ADAMTS13 [53]. These results indicate that the M and D* domains may form a stable association that is not altered during the catalytic cycle and constitute a functional part of the proteinase domain. This is supported by absence of the D* domain in all ADAMTS-L proteins (Figure 1).

The homologous ADAM_CR domains, D* and C_A_, are separated by about 45Å along T1. T1 has a very similar structure to the prototypical TSR, TSR2 in TSP-1 [133] adopting a long, twisted and antiparallel three-stranded fold (Figure 9B). The core of the T1 structure is stabilized by stacked layers of tryptophan, arginine, and hydrophobic residues, and is capped by disulfide bonds at both ends (Cys411/Cys423 and Cys396/Cys433), which has been referred to as the “CWR-layered core” [133]. In addition to the CWR-layered core, the second and third strands in T1 form a regular antiparallel β-sheet, whereas the bulged third strand is stabilized by hydrogen bonds between the side chains of three serine residues (Ser388, Ser394 and Ser397) and backbone nitrogen atoms from the neighboring strand. The residues involved in the CWR-layered core and the serine residues in the bulged strand are highly conserved among the T1 portions of ADAMTS and ADAMTS-L members [53] (Figure 9C). The β-sheet in T1 stacks against the *C*-terminal β-sheet in the C_A_ subdomain, forming a mini β-sandwich structure with a hydrophobic core that strengthens the interactions between T1 and C_A,_ thus fixing the C_A_ domain position relative to T1. On the other hand, there are few specific interactions between the D* and T1 domains in the crystal structure of ADAMTS13-DTCS, suggesting that the relative orientation between the D* and T1 domains may be fixed by crystal packing and would be variable in solution. The flexibility of the molecule between the D* and T1 domains is reflected by the low isomorphism of the ADAMTS13-DTCS crystals [53,134]. The C_B_ subdomain has no apparent secondary structure but comprises a series of turns stabilized by a pair of disulfide bonds and forms a rod shape with its N and C termini about 25Å apart (Figure 9A). The C_A_ and S domains are bridged by the C_B_ subdomain whose amino-acid sequence is highly conserved among ADAMTSs and ADAMTS-Ls [53] (Figure 9C). In the crystal structure of ADAMTS13-DTCS, direct contact exists between the C_A_ domain and the extended loop in the S domain. The mutants with an extra disulfide bond formed between the C_A_ and S domains affected nether secretion nor enzymatic activity, suggesting that the C_A_ and S domains form a stable association and that functional detachment between the domains does not occur during ADAMTS13 function [53]. The residues involved in the interaction between the C_A_ and S domains are conserved among ADAMTS13s from different species, but not among other ADAMTS members. Therefore, whether the stable association between the C_A_ and S domains is conserved in other ADAMTS members remains to be elucidated.

**Figure 10:**
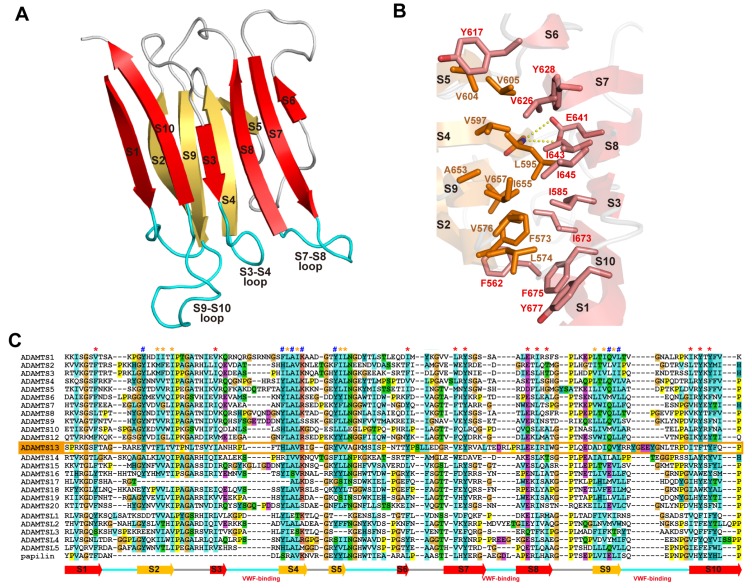
S domain structure. (**A**) Ribbon representation of the crystal structure of the S domain of ADAMTS13. The strands in the two β-sheets are shown in red and orange. (**B**) Close-up view of the hydrophobic core between the β-sheets. Side chains forming the hydrophobic core and the conserved Glu641, whose side-chain oxygen atoms make hydrogen bonds with the backbone nitrogen atom of Leu595 in the opposing strand, are indicated. (**C**) Sequence alignment of the S domain of human ADAMTSs, ADAMTS-L and papilin. The residues in the hydrophobic core and the conserved aromatic surface cluster [53] are marked with * and #, respectively.

The structure of the S domain of ADAMTSs is currently only available for ADAMTS13 [53]. The nomenclature of the “spacer” domain of ADAMTSs comes from the fact that this region is a long cysteine-less segment and its primary structure shows no apparent homology to known structural motifs. However, the crystal structure of the ADAMTS13 S domain and the structure-based sequence alignments revealed that all ADAMTS and ADAMTS-L members share the single globular S domain structure with 10 β-strands in a jelly-roll topology, forming two antiparallel β-sheets that lie almost parallel to each other [53] (Figure 10A). Conserved hydrophobic residues form the core of the β-sandwich (Figure 10B,C), while loops located at the distal end of the molecule are highly variable in both in length and amino acid sequences among ADAMTSs and ADAMTS-Ls (Figure 10C), suggesting these loops could form protein–protein interaction sites. The N and C termini of the S domain lie in close proximity to one another, and thus the T2 domain that follows the S domain should be protruding out from near the C_B_/S-domain junction but not from the distal side of the S domain.

## 4. ADAMTS13 and VWF Interaction

Significant progress in our knowledge of the structure-function relationship of the M12B clan proteinases has been made by studies on ADAMTS13 [135,136], including the demonstration of the actual involvement of the ADAM_CR and S domains in substrate recognition by intensive mutagenesis experiments.

**Figure 11:**
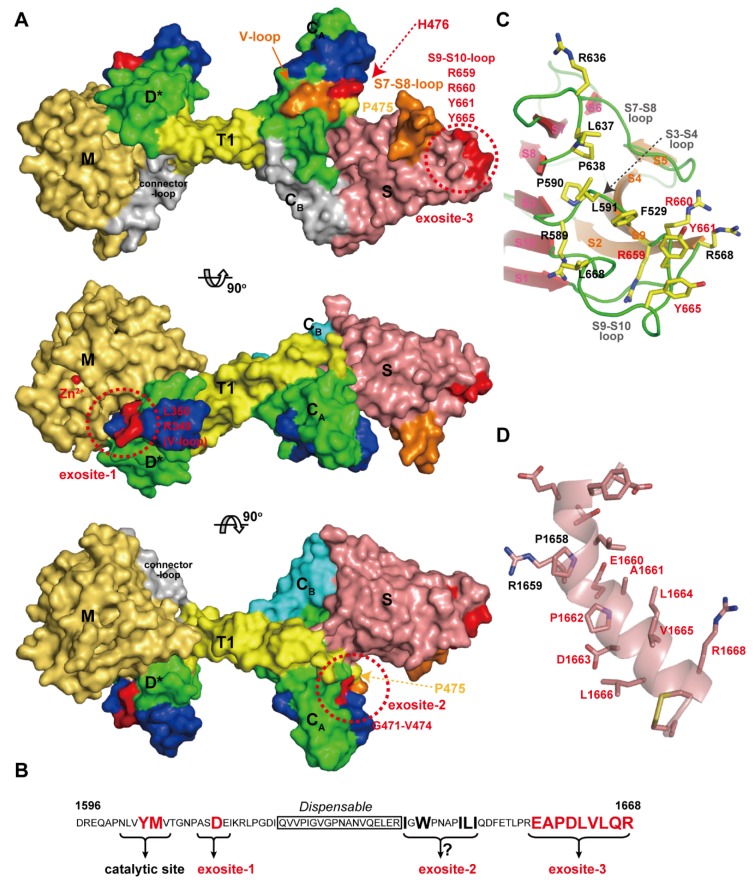
ADAMTS13 and VWF interaction. (**A**) Models of the ADAMTS13-MDTCS shown at 90 degree rotations. The residues important for the interaction with VWF are shown in red and other potential VWF-binding sites are shown in orange. Domains are colored as in Figure 7A. (**B**) Amino acid sequence of VWF (D1596-R1668) and the ADAMTS13-interacting sites are schematically represented. (**C**) Close-up view of the exosite-3 in the S domain, the hydrophobic cluster surrounded by arginine residues. The residues important for VWF-binding are indicated with red letters. (**D**) Close-up view of the VWF segment (D1653-C1670) in an α-helical conformation observed in the crystal structure of the VWF A2 domain [153]. The figure was created in reference to the original drawing by de Groot *et al.* [152].

Von Willebrand Factor (VWF) is a plasma glycoprotein that plays an essential role in platelet dependent hemostasis [137,138]. VWF (2050 amino acid residues) circulates in blood in multimeric forms of highly variable size, ranging from dimers to species that may exceed 60-mers (UL-VWF multimers) [139]. In healthy individuals, UL-VWF multimers undergo limited proteolytic processing by ADAMTS13 [18]. Deficiency in ADAMTS13 activity either by genetic mutations in the ADAMTS13 gene or by acquired inhibitory autoantibodies directed against the ADAMTS13 protein, result in the accumulation of UL-VWF in the plasma. UL-VWF accumulation leads to the formation of disseminated platelet-rich micro thrombi in the micro-vasculature, which results in the life-threatening disease TTP [17,18,19,140,141]. ADAMTS13 specifically cleaves the Tyr1605-Met1606 peptidyl bond within the A2 domain of VWF [142] in a fluid shear-stress-dependent manner [143,144]. The MD*TCS domains of ADAMTS13 (ADAMTS13-MD*TCS) are necessary and sufficient for specific proteolytic cleavage of VWF *in vitro* [145,146,147,148]. VWF73 (residues 1595–1668 in the VWF A2 domain) was identified as a minimum specific substrate for ADAMTS13 and suggested that a segment (residues 1607–1668) of VWF73 contains essential residues for recognition by ADAMTS13 [149]. Recent studies have added to our understanding of this recognition, revealing that specific regions in ADAMTS13, namely exosites-1, -2 and -3, in the D*, C_A_ and S domains respectively, are all required for its interaction with VWF. These exosites of ADAMTS13 directly interact in a linear fashion with various segments in the central VWF-A2 domain between residues Ala1612 and Arg1668. In addition, fine mapping of epitopes of anti-ADAMTS13 antibodies derived from TTP patients, has provided further insight into the structural elements in ADAMTS13 that are essential for VWF binding. Figure 11A represents a summary of our understanding of the VWF-interacting sites in ADAMTS13 mapped on the molecular surface. Corresponding ADAMTS13-binding sites within VWF (residues 1596–1668) are schematically indicated in Figure 11B.

The segment corresponding to the HVR runs across the middle of the D* domain of ADAMTSs in close proximity to the active site, suggesting that the HVR might be ideally positioned to directly influence cleavage of the substrate [44]. In the D* domain in ADAMTS13, the HVR, together with the V-loop located beside it, was shown to form part of exosite I. ADAMTS13 variants carrying a point mutation, R349A or L350G [150], or R349D [53] in the HVR or a deletion of the V-loop (residues 324–330) [53] displayed a dramatically reduced proteolytic activity. Further studies demonstrated that residues Arg349 and Leu350 of the D domain of ADAMTS13 may interact with residues Asp1614 and Ala1612, respectively, in the central A2 domain of VWF [150]. These interactions, in addition to the direct active site cleft interactions in the M domain, may help orientate the scissile bond toward the active site center of ADAMTS13 [151].

The C_A_ subdomain adopts an ADAM_CR domain fold and thus potentially functions as a protein–protein interaction site. As expected, ΔV-loop, a triple alanine substitution (H476A/S477A/Q478A) in the V-loop and R488E in the HVR mutants had significantly reduced proteolytic activity, suggesting that these hydrophilic or charged residues play a pivotal role in VWF recognition and constitute exosite-2 [53]. Recently, de Groot *et al.*, reported the results of a comprehensive analysis of the C domain in ADAMTS13 that identified its functional importance for interacting with VWF [152]. They found that mutagenesis of the 11 predominantly-charged residues in the C domain (actually in the C_A_ subdomain) had no major effect on ADAMTS13 function, and five out of six engineered glycans on the C domain also had no effect on ADAMTS13 function. However, glycans attached at position 476 appreciably reduced both VWF binding and proteolysis.

By substituting the segments of the C domain with the corresponding regions in ADAMTS1, they identified that residues Gly471-Val474 at the base of the V-loop within the C_A_ subdomain form a hydrophobic pocket that appears to be involved in binding hydrophobic residues Ile1642, Trp1644, Ile1649, Leu1650 and Ile1651 in VWF. The east Asian-specific P475S polymorphism in the ADAMTS13 gene causes approximately 16% reduction in plasma ADAMTS13 activity [154]. The crystal structure of ADAMTS13-DTCS (P475S) revealed that the conformation of the V-loop in the C_A_ subdomain of this mutant was significantly different from that of the wild type [73].

The S domain in ADAMTS13 has the highest binding affinity for the A2 site of VWF. *C*-terminal deletion mutants of the VWF115 (VWF residues 1554–1668) and VWF73 fragments demonstrated that VWF A2 domain residues Glu1660-Arg1668 appreciably contribute to the cleavage of the Try1605-Met1606 scissile bond [149] and that the S domain of ADAMTS13 binds to this sequence [155]. As previously mentioned, the distal loops in the S domain are highly variable among ADAMTS/ADAMTS-L members (Figure 10C) and are thus suggested to create a substrate-binding exosite [53]. Mutants in which the S7-S8-loop (residues 606–611) and S9-S10-loop were replaced by short linkers, showed greatly reduced enzymatic activity for FRET-VWF73 [53]. In ADAMTS13, these variable loops create a hydrophobic cluster that is surrounded by arginine residues (Figure 11C). Systematic site-directed mutagenesis identified that this hydrophobic cluster rimmed with arginine residues actually constitutes another VWF-binding exosite (exosite-3) [53], and further identified Arg659, Arg660 and Tyr661 as critical residues for VWF cleavage [156]. It was also demonstrated that Arg660, Tyr661, and Tyr665 in the S domain of ADAMTS13 represent a core binding site for autoantibodies isolated from patients with acquired TTP [157]. The ADAMTS13 variants, R600K/F592Y/R568K/Y661F and R660/F592Y/R568K/Y661/Y665F, exhibit increased specific activity for both peptide substrates and multimeric VWF [158]. These gain-of-function ADAMTS13 variants were more resistant to inhibition by autoantibodies from idiopathic TTP patients because of reduced binding by anti-ADAMTS13 IgGs [158]. Both the surface properties and the size of exosite-3 imply that it binds to VWF, such that the VWF segment (residues 1653–1668) forms an amphiphilic α-helix (Figure 11D) and makes contact with ADAMTS13 by facing its hydrophobic surface toward exosite-3 [53]. Similar to ADAMTS13, the removal of the S domain dramatically reduces the aggrecanaolytic activity of ADAMTS5 and further removal of the C domain essentially abolished the activity [159]. An antibody reacting with the S domain of ADAMTS5 was shown to block the cleavage of aggrecan by the enzyme [160]; however, the exact site of ADAMTS5 that reacts with the antibody has not been identified.

## 5. Concluding Remarks

Tremendous progress has been made in the past decade towards our understanding of the structure–function relationship of the M12B clan of proteinases. Crystallographic studies have revealed the structures and spatial relationships of the functionally important domains of both ADAM and ADAMTS family proteinases. Most of the structural information of the overall MDC domains of ADAMs has come from SVMPs. The higher abundance, stability and resistance to proteolysis of SVMPs compared to mammalian ADAMs have made them attractive models for structural studies. The key message from these findings is that the MDC domains adopt a C-shaped configuration, whereby the HVR in the ADAM_CR domain faces toward the catalytic site. This raises the intriguing possibility that the HVR creates an exosite for capturing substrates directly or via binding to an associated protein. The RVV-X structure is consistent with this hypothesis. The multactivase structure suggests a potential function of the D domain in protein–protein interactions. These structural studies on SVMPs have provided radical new insights into the structure–function relationship of ADAMs. Some molecules have been shown to work as cofactors in the process of ectodomain shedding by membrane-bound ADAMs [161,162,163]; however, how these molecules function with ADAMs at a molecular level remains to be elucidated. Moreover, fundamental aspects of the functions of ADAMs, such as how membrane-bound ADAMs select their substrate and how their activity is regulated, are still largely unknown. A crystal structure of ADAM in complex with a substrate and/or such a cofactor would greatly improve our understanding of ADAMs’ functions. Recent advances in ADAMTS13 research have provided invaluable information not only for our understanding of the mechanisms underlying TTP but also for designing structure-function studies for other family members. Notably, ADAMTSs contain no disintegrin-like structures but instead have two ADAM_CR domains that actually constitute VWF-binding exosites in ADAMTS13. This finding strongly supports the idea that the ADAM_CR domain functions as a novel protein-protein interaction module. The S domain, uniquely found in ADAMTSs and ADAMTS-Ls among the 12B members, may also provide another protein-protein interaction site for these members. The functions of the distal domains, which are variable among ADAMTS members, are still largely unknown. Recently, the distal T2-CUB2 domains were shown to directly interact with the proximal MD*TCS domains and inhibit substrate cleavage, while binding of VWF to the distal ADAMTS13 domains relieves this autoinhibition. Thus, ADAMTS13 is regulated by substrate-induced allosteric activation [164,165]. Whether the distal domains of other ADAMTSs have similar allosteric properties or not remains to be determined. The growing number of links with human diseases makes ADAMs and ADAMTSs attractive targets for novel therapies. To date, no successful treatment exists involving specific ADAM/ADAMTS inhibitors targeting the catalytic site. MMPs were also considered valuable therapeutic targets; however, early trials of small-molecule inhibitors (SMIs) toward their catalytic site failed due to poor inhibitor specificity profiles [166]. Because of the structural similarity of the catalytic sites of MMPs and ADAMSs/ADAMTSs, there is a limitation in generating active-site-targeted SMIs that are selective to one metalloproteinase species. Although we still have limited knowledge of how the prodomain controls enzymatic activity because of a lack of crystal structures, recombinant prodomains of ADAMs can act as inhibitors and might be used as alternatives to SMIs [167,168]. A unique cross-domain inhibitory antibody against ADAM17 has also been proposed [169]. Exosite or allosteric inhibitors may have more advantages in increasing the selectivity against specific ADAMs/ADAMTSs. Recently, three groups reported antibody-based exosite inhibitors of ADAMTS5, which were generated for therapeutic purposes to protect the destruction of articular cartilage in osteoarthritis [160,170,171]. Further structural knowledge of the exosite interactions of ADAM/ADAMTS family proteinases and their substrates will facilitate the development of novel inhibitors that may block cleavage of specific substrates, while leaving other catalytic functions of the targeted enzyme unaltered.

## Figures and Tables

**Table 1:** Selection of the 3D structures of the M12B proteinases deposited in the PDB.

Protein	Source	Domains	PDB ID	Year	Reference	
**ADAMs**					
ADAM8	human	M	4DD8	2012	[68]
ADAM10	bovine	DC	2AO7	2005	[48]
ADAM17	human	M	1BKC	1998	[64]
ADAM17	human	C	2M2F (NMR)	2013	[69]
ADAM22	human	MDCE	3G5C	2009	[65]
ADAM33	human	M	1R54, 1R55	2004	[70]
**ADAMTSs**					
ADAMTS1	human	MD*	2JIH, 2V4B	2007	[66]
ADAMTS4	human	MD*	2RJP, 3B2Z	2008	[71]
ADAMTS5	human	M	3B8Z	2008	[72]
ADAMTS5	human	MD*	2RJQ	2008	[71]
ADAMTS13	human	D*TCS	3GHM, 3GHN, 3VN4	2009	[53,73]
**P-I SVMPs**					
acutolysin A	*A. acutus*	M	1BSW, 1BUD	1998	[74]
acutolysin C	*A. acutus*	M	1QUA	1999	[75]
adamalysin II	*C. adamantus*	M	1IAG	1993	[42,76]
atrolysin C	*C. atrox*	M	1ATL, 1HTD	1994	[77]
BaP1	*B. asper*	M	1ND1	2003	[78]
BmooMPα-I	*B. moojeni*	M	3GBO	2010	[79]
F II	*A. acutus*	M	1YP1	2005	[80]
H2 proteinase	*T. flavoviridis*	M	1WNI	1996	[81]
TM-1	*T. mucrosquamatus*	M	4J4M	2013	[82]
TM-3	*T. mucrosquamatus*	M	1KUF, 1KUG, 1KUI, 1KUK	2002	[83]
**P-IIIa/b SVMPs**					
AaHIV	*A. acutus*	MDC	3HDB	2009	[84]
atragin	*N. atra*	MDC	3K7L	2010	[85]
bothropasin	*B. jararaca*	MDC	3DSL	2008	[86]
catrocollastain/VAP2B	*C. atrox*	MDC	2DW0, 2DQ1, 2DW2	2007	[87]
K-like	*N. atra*	MDC	3K7N	2010	[85]
**P-IIIc SVMPs**					
VAP1	*C. atrox*	2x(MDC)	2ERO, 2ERP, 2ERQ	2006	[28]
**P-IIId SVMPs**					
RVV-X	*D. russelli*	MDC+snaclec	2E3X	2007	[88]
multactivase	*E. multisquamatus*	DC+snaclec		unpublished	

* Despite its name, the D domain of ADAMTSs actually does not adopt a classic “disintegrin-like” tertiary structure, but has an ADAM_CR domain fold and is thus indicated as “D*”.

## Abbreviations

The following abbreviations are used in this manuscript:

ADAM: a disintegrin and metalloproteinase
ADAMTS: a disintegrin-like and metalloproteinase with thrombospondin type-1 motif
ADAMTS-L: ADAMTS-like proteins
EGF: epidermal growth factor
Gla: γ-carboxyglutamic acid
MMP: matrix metalloproteinase
PDI: protein-disulfide isomerase
RVV-X: Russell’s viper venom factor *X* activator
SMI: small-molecule inhibitor
Snaclec: snake venom C-type lectin like protein
SVMP: snake venom metalloproteinase
TSP: thrombospondin
TSR: thrombospondin type-1 repeat
TTP: thrombotic thrombocytopenic purpura
VAP1: vascular apoptosis inducing protein-1
VWF: von Willebrand Factor
